# Association of metabolic score for insulin resistance with progression or regression of prediabetes: evidence from a multicenter Chinese medical examination cohort study

**DOI:** 10.3389/fendo.2024.1388751

**Published:** 2024-11-11

**Authors:** Guotai Sheng, Maobin Kuang, Ruijuan Yang, Yang Zou

**Affiliations:** ^1^ Jiangxi Provincial Geriatric Hospital, Jiangxi Provincial People’s Hospital, The First Affiliated Hospital of Nanchang Medical College, Nanchang, Jiangxi, China; ^2^ Jiangxi Medical College, Nanchang University, Nanchang, Jiangxi, China; ^3^ Jiangxi Cardiovascular Research Institute, Jiangxi Provincial People’s Hospital, The First Affiliated Hospital of Nanchang Medical College, Nanchang, Jiangxi, China; ^4^ Department of Endocrinology, Jiangxi Provincial People’s Hospital, The First Affiliated Hospital of Nanchang Medical College, Nanchang, Jiangxi, China

**Keywords:** diabetes, metabolic score for insulin resistance, progression of prediabetes, regression of prediabetes, Chinese

## Abstract

**Objective:**

Few studies have evaluated the changes in blood glucose status in individuals with prediabetes, and this study aimed to analyze the association between metabolic score for insulin resistance (MetS-IR) and the progression or regression of prediabetes.

**Methods:**

This retrospective cohort study used research data from medical examination institutions under the Rich Healthcare Group in 32 regions across 11 cities in China. Progression of prediabetes to diabetes and regression to normal fasting glucose (NFG) were defined based on glycemic changes during follow-up. The association between MetS-IR and the progression or regression of prediabetes was analyzed using multivariate Cox regression, restricted cubic splines, and piecewise regression models.

**Results:**

Data from 15,421 prediabetic subjects were analyzed. Over an average follow-up of 2.96 years, 6,481 individuals (42.03%) returned to NFG, and 2,424 (15.72%) progressed to diabetes. After controlling for confounding factors, an increase in MetS-IR was observed to increase the risk of diabetes onset in the prediabetic population, whereas a decrease in MetS-IR had a protective effect for returning to NFG. Additionally, a nonlinear relationship between MetS-IR and prediabetes regression was observed, with 37.22 identified as the inflection point; prediabetes regression rates were significantly higher before this point and markedly decreased after it.

**Conclusion:**

For individuals with prediabetes, an increase in MetS-IR may lead to an increased risk of diabetes; conversely, a decrease in MetS-IR enhances the protective effect for returning to NFG and keeping MetS-IR below 37.22 is significant for the regression of prediabetes.

## Introduction

Prediabetes is an intermediate state defined by blood glucose levels that are below the threshold for diabetes but above normal glucose levels (1). Currently, this intermediate state directly affects over 400 million people globally, and it is projected that by 2045, over 600 million people will be in this state (2). Prediabetes, as the term suggests, is a necessary phase for the majority of diabetes patients. However, recent studies have found that prediabetes not only plays a significant role in the onset of diabetes (1–4), but it also substantially increases the risk of vascular-related diseases, neurological disorders, cancer, kidney diseases (5–7), and is closely associated with accelerated brain aging and bone loss (8, 9), even significantly impacting life expectancy (8). These findings emphasize the widespread adverse effects of prediabetes on health; fortunately, the prediabetic state offers considerable potential for reversing blood glucose regulation back to normal and significantly reducing the risk of diabetes and its complications. Randomized controlled trials have shown that approximately 32.2%-52.1% of individuals with prediabetes return to normal blood glucose levels in the short term through intensified lifestyle or pharmacological interventions, and these remissions and benefits continue to increase over time (10–21). Therefore, from a public health perspective, early identification and monitoring of important modifiable factors that can affect the progression and regression of prediabetes are crucial for diabetes prevention and intervention.

Insulin resistance (IR) is one of the most important pathophysiological characteristics of both prediabetes and diabetes (1, 3, 4), and early monitoring and control of IR are helpful for the regression of prediabetes (22–24). The hyperinsulinemic-euglycemic clamp (HEGC), developed in the 1970s, has long been the gold standard for measuring IR (25). However, with the rapid development of epidemiology, large-scale population screening and monitoring have become more common, making HEGC less suitable for epidemiological screening due to its relative complexity and invasiveness. To address this, researchers have developed various simple and convenient surrogate measures of IR to replace HEGC (26). Metabolic score for insulin resistance (MetS-IR), proposed by Professor Bello-Chavolla OY and his team, is a promising new surrogate measure of IR (27). In initial studies, Bello-Chavolla OY et al. compared the diagnostic performance of MetS-IR against the M-value adjusted by fat-free mass obtained by the HEGC and found that MetS-IR had a better correlation with detecting IR compared to HEGC. Subsequent studies further confirmed the significant value of MetS-IR in predicting and assessing the risk of diabetes (28, 29). Recently, we also evaluated the effects of multiple IR surrogates including triglycerides glucose index, triglycerides glucose-body mass index, triglycerides/high-density lipoprotein cholesterol (TG/HDL-C) ratio and MetS-IR on diabetes progression based on two independent cohorts in China and Japan (30); Our preliminary study found that MetS-IR is the best IR surrogate for Chinese and Japanese populations in terms of risk assessment and prediction of diabetes progression. However, the association of MetS-IR with the progression or regression of prediabetes remains unclear. Given the modifiable nature of the prediabetic state, clarifying the relationship between the IR surrogate MetS-IR and the progression or regression of prediabetes early on has significant clinical value for early intervention and reversal of prediabetes. To address this issue, in this study, we aimed to determine the relationship between MetS-IR and the progression or regression of prediabetes.

## Methods

### Data source

All data for this study were sourced from a multicenter health examination cohort of the Rich Healthcare Group in China. The multicenter retrospective cohort study, composed of a general examination population, aimed to explore risk factors influencing the onset of diabetes. The available dataset was organized and uploaded to the Dryad database by Professor Chen and his team for public sharing (31). According to the Dryad Data Sharing Terms and the Creative Commons Attribution-NonCommercial-ShareAlike 4.0 International License, researchers are permitted to use the datasets publicly shared in the Dryad database for secondary creation, following the proper citation of the data source (31).

### Study design

The Rich Healthcare Group multicenter health examination cohort was established by Professor Chen and his team. It includes adults (685,277) who had at least two health examinations at Rich Healthcare Group facilities across 32 areas in 11 Chinese cities from 2010 to 2016. Initially, Chen and colleagues aimed to explore the impact of the obesity measurement parameter, body mass index (BMI), on diabetes onset (32). Based on the research objectives, they excluded subjects with: (i) a clear diabetes diagnosis at baseline or diabetic status could not be determined during subsequent visits (6,630 subjects); (ii) less than a 2-year interval between two visits (324,233 subjects); (iii) a BMI less than 15 kg/m² or higher than 55 kg/m² at baseline (152 subjects); (iv) missing age, gender, and fasting plasma glucose (FPG) data at baseline (135,317 subjects). Ultimately, 211,833 subjects were included in Chen et al.’s study.

The current study, using the shared dataset (31) provided by Chen and colleagues, aimed to further explore the association between the IR surrogate MetS-IR and the progression or regression of prediabetes. Based on the inclusion and exclusion criteria established by Chen et al., we further excluded subjects with: (i) non-prediabetes status at baseline (185,815 subjects); (ii) missing MetS-IR data at baseline (10,594 subjects); (iii) missing FPG measurements during follow-up (3 subjects). Finally, our study included 15,421 subjects for analysis.

### Ethical approval

As a secondary analysis, the current study’s protocol was submitted to and approved by the Ethics Committee of Jiangxi Provincial People’s Hospital. Considering that this research is a secondary creation based on completed studies and the dataset is anonymized, the Committee waived the requirement for informed consent from each study participant.

### Data collection and measurement

During each visit to the examination centers, each participant, assisted by medical staff, recorded their diabetes history, family history of diabetes, personal lifestyle information (drinking/smoking status), demographic information (gender and age), and basic physical measurement parameters (blood pressure, height, and weight). Blood pressure was measured using a sphygmomanometer, recording systolic blood pressure (SBP) and diastolic blood pressure (DBP). Height and weight were measured without shoes and in light clothing, recorded to 0.001m and 0.1kg precision, respectively, and used to calculate each individual’s BMI.

Biochemical blood measurements were conducted on venous blood drawn after at least 10 hours of fasting. In standard laboratories, professional technicians measured total cholesterol (TC), TG, HDL-C, low-density lipoprotein cholesterol (LDL-C), FPG, creatinine (Cr), blood urea nitrogen (BUN), alanine aminotransferase (ALT), and aspartate aminotransferase (AST) using an automatic analyzer (Beckman 5800).

Calculation of MetS-IR: $\text{Ln }\left[\left(2\;\times \;\text{FPG }\left(\text{mg}/\text{dL}\right)\right)\;+\;\text{TG }\left(\text{mg}/\text{dL}\right)\right]\;\times \;\text{BMI}\;\left(\text{kg}/{\text{m}}^{2}\right)/\left(\text{Ln }\left[\text{HDL-C}\;\left(\text{mg}/\text{dL}\right)\right]\right)$ (27).

### Assessment of study outcomes

The study outcomes were primarily categorized into three groups: regression of prediabetes, maintenance of prediabetes status, and progression to diabetes. Based on the American Diabetes Association’s criteria for prediabetes/diabetes using FPG, we defined the following study outcomes (33): (i) regression of prediabetes defined as an FPG measurement less than 5.6 mmol/L during follow-up; (ii) maintenance of the prediabetic state defined as FPG levels between 5.6 and 6.9 mmol/L during follow-up. (iii) progression to diabetes defined as an FPG measurement above 7.0 mmol/L during follow-up or self-reported diagnosis of diabetes by other healthcare professionals.

### Statistical analysis

Participants were categorized according to the study outcomes and their baseline characteristics were compared using univariate analysis of variance/Chi-square test/Kruskal-Wallis H test. Baseline information results were presented as median (interquartile range)/mean (standard deviation)/frequency (%).

For the three-category study outcomes, we considered participants who continued to maintain prediabetes status during follow-up as the non-event group and used a one-to-one method to split the data into binary datasets with prediabetes regression or progression as outcomes (34, 35). The association between MetS-IR and the progression or regression of prediabetes was analyzed using Cox regression models. Prior to model establishment, we assessed the collinearity of MetS-IR with other covariates using prediabetes progression or regression as outcome variables (**Supplementary Tables 1**, **2**), as well as the Schoenfeld residuals plots of MetS-IR over time (**Supplementary Figures 1**, **2**) (36, 37). After confirming that the Cox models met the proportional hazards assumption and identifying weight, BMI, and TC as collinear variables, we constructed four differently adjusted Cox regression models according to the STROBE statement: The unadjusted model was first constructed, analyzing the association between MetS-IR and the progression or regression of prediabetes without adjusting for any variables; then, Model I adjusted for demographic and lifestyle factors (age, gender, height, family history of diabetes, smoking status, and drinking status); Model II further considered blood pressure, blood glucose, and blood lipid-related factors (SBP, DBP, FPG, TG, HDL-C, and LDL-C) based on Model I; Model III, the final model, additionally considered liver and kidney function impacts (ALT, BUN, Cr). On the basis of adjusting covariates in Model III, we also plotted dose-response curves of MetS-IR with prediabetes regression or progression using 4-knot restricted cubic splines (RCS) regression model. When nonlinear associations were detected, potential inflection point values were calculated using recursive algorithms, and segmented Cox regression was used to assess HR values for prediabetes progression or regression before and after the inflection point.

Additionally, several sensitivity analyses were conducted: (i) To further verify the validity of the association between MetS-IR and prediabetes progression or regression, we treated the continuous variable MetS-IR as a categorical variable based on its quintiles and conducted similar analyses. (ii) According to the World Health Organization (WHO) standards for prediabetes/diabetes based on FPG (38), we analyzed the association between MetS-IR and prediabetes progression or regression in a new study population. (iii) We further validated the aforementioned associations by applying models with diabetes progression and return to normal fasting glucose (NFG) as competing risks. (iv) Considering the indirect influence of family history of diabetes (39), we selected a study population without a family history of diabetes and repeated the above analyses. (v) To verify the appropriateness of the dose-response curves between MetS-IR and prediabetes progression or regression, we continued using 3- and 5-knot additional analyses to test the stability of RCS results.

We also conducted stratification to further explore whether the association between MetS-IR and prediabetes progression or regression existed in potential specific populations, based on age (according to WHO age division standards) (40), gender, and BMI (according to the Chinese Obesity Working Group BMI division standards) (41), and used likelihood ratio tests to detect whether this association was significantly different between stratifications.

All analyses were performed using R language version 4.2.1 and Empower(R) version 2.20 statistical software. A two-sided *p*-value of less than 0.05 was considered statistically significant.

## Results

### Follow-up results on progression or regression of prediabetes

**Figure 1:**
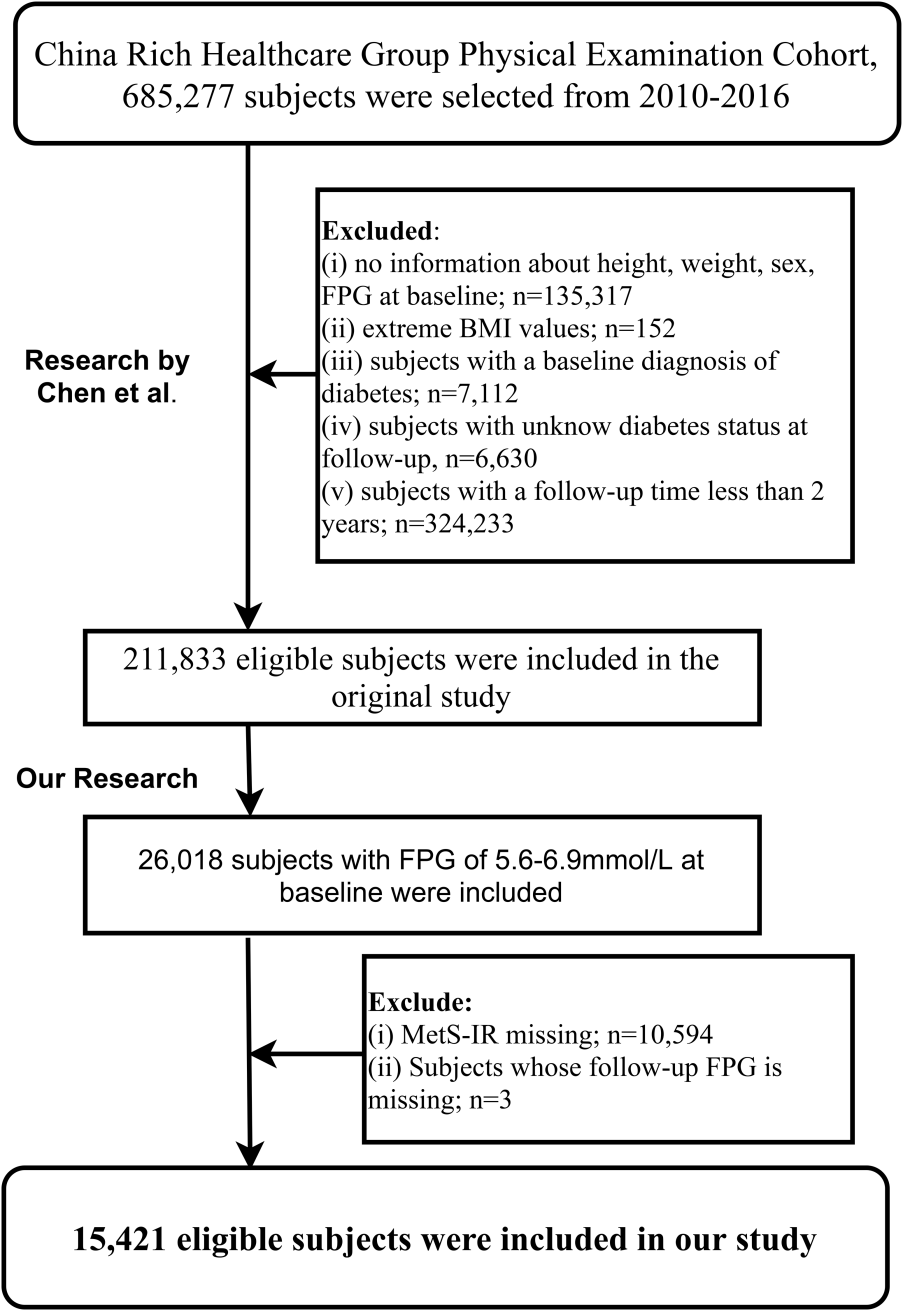
Flow chart of study participants.

**Figure 2:**
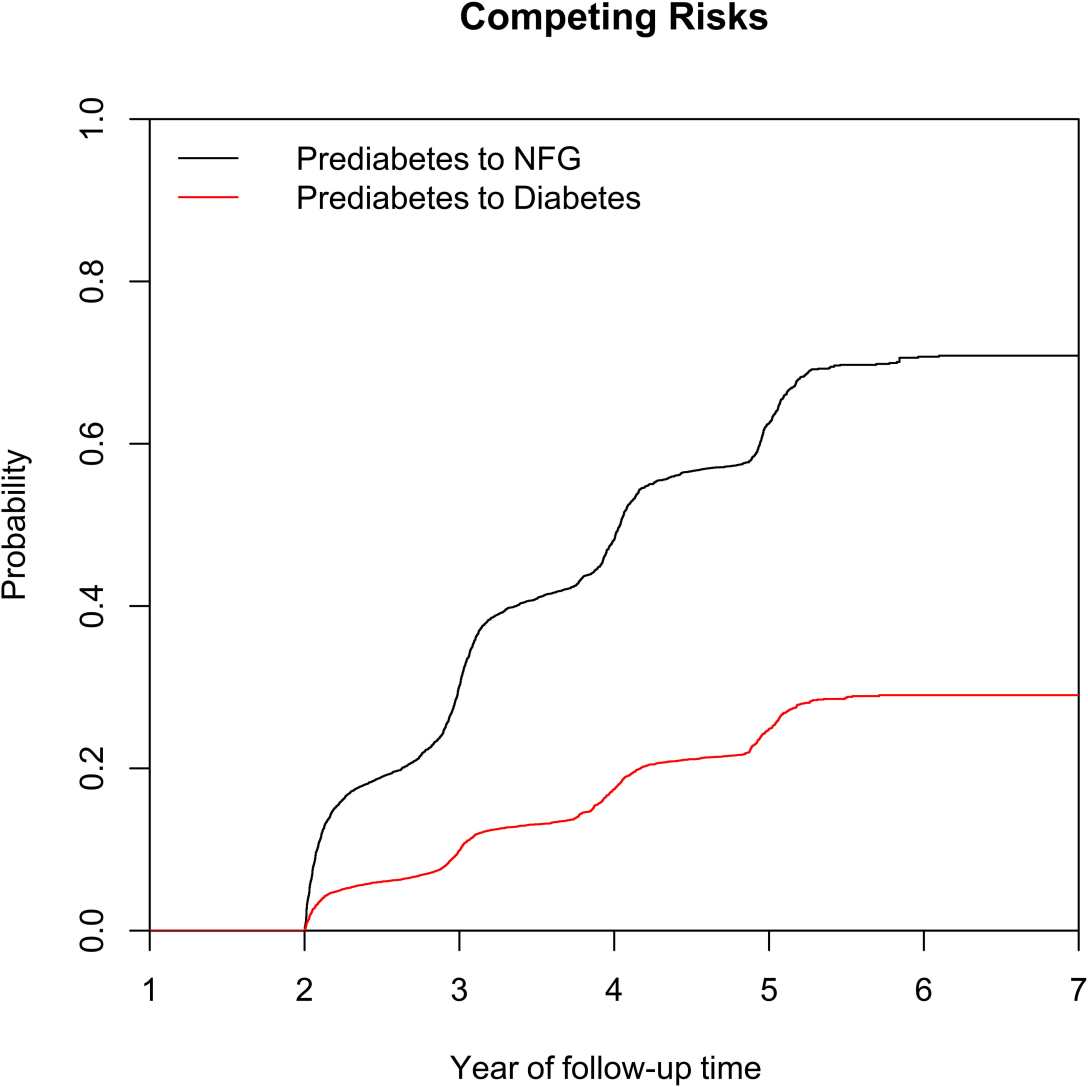
Cumulative incidence curve of prediabetes recovering to NFG or progressing to diabetes. NFG: normal fasting glucose.

**Figure 1** illustrates the selection process of the study population. A total of 15,421 eligible prediabetic subjects were included in this study, with an average age of 50 years and a male-to-female ratio of 1.85:1. During the average follow-up period of 2.96 years, 6,481 individuals (42.03%) returned to NFG, 2,424 (15.72%) progressed to diabetes, and 6,481 (42.03%) continued to maintain their prediabetic status. The cumulative incidence function calculated the cumulative risk of prediabetes progression or regression, showing that the cumulative incidence rates of prediabetes progression or regression at the third, fourth, fifth, and sixth years were 9.86%/30.11%, 17.46%/48.22%, 24.86%/62.51%, and 29.02%/70.72%, respectively. **Figure 2** displays the cumulative incidence curves of prediabetes progression or regression, indicating a higher adjustability for regression to NFG compared to progression to diabetes.

### Characteristics of the study population at baseline retrospectively summarized based on follow-up results

**Figure 3:**
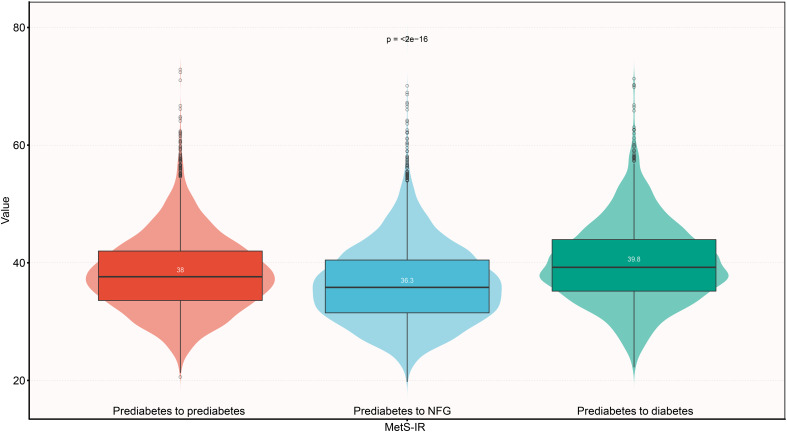
Violin chart showing baseline valus of MetS-IR according to glucose status during follow-up. Mets-IR: metabolic score for insulin resistance; NFG: normal fasting glucose.

We reviewed study characteristics of participants at baseline according to study outcomes at follow-up (**Table 1**). Reports indicated that female participants and those who quit smoking and drinking were more likely to return to NFG in the future. Additionally, subjects who returned to NFG typically had lower age, weight, BMI, SBP, DBP, FPG, TC, TG, LDL-C, ALT, AST, BUN, Cr levels at baseline. In addition, it is worth noting that among the subjects, those who reverted to NFG and those who later progressed to diabetes had the lowest and highest baseline MetS-IR, respectively (**Figure 3**); This result suggested that high MetS-IR may be a factor promoting the occurrence of diabetes, while low MetS-IR may be an important factor in promoting NFG recovery.

**Table 1:** Baseline characteristics summarized according to subjects' glycemic status during follow-up.

	Glucose status during follow-up			*P*-value
	Prediabetes	NFG	Diabetes	
No. of subjects	6516	6481	2424	
Sex				<0.001
Male	4358 (43.54%)	3949 (39.45%)	1702 (17.01%)	
Female	2158 (39.87%)	2532 (46.78%)	722 (13.34%)	
Age, years	53.00 (43.00-62.00)	46.00 (36.00-58.00)	55.00 (46.00-63.00)	<0.001
Height, cm	166.59 (8.37)	166.71 (8.40)	166.74 (8.31)	0.625
Weight, kg	69.80 (11.81)	67.64 (12.12)	72.22 (12.60)	<0.001
BMI, kg/m^2^	25.06 (3.19)	24.23 (3.28)	25.87 (3.41)	<0.001
SBP, mmHg	129.35 (17.76)	124.16 (16.93)	131.46 (17.99)	<0.001
DBP, mmHg	79.53 (11.24)	76.73 (10.84)	80.49 (11.31)	<0.001
FPG, mmol/L	6.00 (0.32)	5.84 (0.24)	6.15 (0.38)	<0.001
TC, mmol/L	5.08 (0.94)	4.99 (0.94)	5.10 (0.97)	<0.001
TG, mmol/L	1.50 (1.04-2.20)	1.31 (0.90-1.97)	1.67 (1.14-2.45)	<0.001
HDL-C, mmol/L	1.31 (1.13-1.51)	1.34 (1.15-1.54)	1.29 (1.09-1.50)	<0.001
LDL-C, mmol/L	2.90 (2.46-3.36)	2.84 (2.41-3.33)	2.88 (2.43-3.40)	<0.001
MetS-IR	38.04 (6.42)	36.29 (6.65)	39.83 (6.90)	<0.001
ALT, U/L	22.50 (16.00-33.00)	20.40 (14.50-31.00)	24.90 (17.80-37.00)	<0.001
AST, U/L	24.00 (20.00-28.80)	23.50 (19.40-28.80)	25.00 (20.70-31.00)	<0.001
BUN, mmol/L	4.91 (4.19-5.78)	4.80 (4.05-5.68)	4.93 (4.13-5.80)	<0.001
Cr, umol/L	73.50 (62.10-84.00)	72.00 (59.80-82.90)	73.30 (62.00-83.00)	<0.001
Family history of diabetes	151 (39.32%)	148 (38.54%)	85 (22.14%)	0.002
Smoking status				<0.001
Current	577 (44.04%)	491 (37.48%)	242 (18.47%)	
Past	94 (36.29%)	121 (46.72%)	44 (16.99%)	
Never	1471 (40.93%)	1642 (45.69%)	481 (13.38%)	
Not recorded	4374 (42.64%)	4227 (41.21%)	1657 (16.15%)	
Drinking status				0.005
Current	100 (46.51%)	86 (40%)	29 (13.49%)	
Past	342 (37.71%)	429 (47.30%)	136 (14.99%)	
Never	1700 (42.07%)	1739 (43.03%)	602 (14.90%)	
Not recorded	4374 (42.64%)	4227 (41.21%)	1657 (16.15%)	

Values were expressed as mean (standard deviation) or medians (quartile interval) or n (%).

Mets-IR, metabolic score for insulin resistance; NFG, normal fasting glucose; BMI, body mass index; SBP, systolic blood pressure; DBP, diastolic blood pressure; FPG, fasting plasma glucose; TG, triglyceride; TC, total cholesterol; HDL-C, high-density lipoprotein cholesterol; LDL-C, low-density lipoprotein cholesterol; ALT, alanine aminotransferase; AST, aspartate aminotransferase; BUN, blood urea nitrogen; Cr, creatinine.

### Association between MetS-IR and progression or regression of prediabetes

The estimated association between MetS-IR (as a continuous variable) and the progression or regression of prediabetes from Cox regression models was displayed in **Table 2**. From four models with different adjustment levels, we found a significant negative correlation between continuous MetS-IR and prediabetes regression, and a positive correlation with prediabetes progression. According to the final model (Model III), each unit increase in MetS-IR resulted in a 26% increased risk of diabetes in prediabetic patients [HR: 1.26, 1.19-1.33], while a decrease in MetS-IR was protective for prediabetic patients (increased probability of returning to NFG), quantified as a 10% protective effect for each unit decrease in MetS-IR [HR: 0.90, 1.19-1.33].

**Table 2:** Multivariate Cox regression analysis of the role of MetS-IR in assessing changes in glycemic status in patients with prediabetes.

	No. of case	HR (95%CI)			
		Non-adjusted Model	Model I	Model II	Model III
Prediabetes to NFG					
MetS-IR		0.82 (0.80, 0.84)	0.85 (0.83, 0.88)	0.89 (0.86, 0.92)	0.90 (0.86, 0.93)
MetS-IR (quintile)					
Q1	1740	Ref	Ref	Ref	Ref
Q2	1396	0.83 (0.78, 0.90)	0.91 (0.84, 0.97)	0.94 (0.87, 1.01)	0.93 (0.87, 1.01)
Q3	1221	0.72 (0.67, 0.77)	0.80 (0.74, 0.86)	0.86 (0.79, 0.93)	0.87 (0.80, 0.95)
Q4	1133	0.67 (0.62, 0.72)	0.76 (0.71, 0.82)	0.83 (0.76, 0.91)	0.84 (0.76, 0.92)
Q5	991	0.58 (0.54, 0.63)	0.64 (0.59, 0.70)	0.74 (0.67, 0.82)	0.76 (0.68, 0.84)
P-trend		<0.0001	<0.0001	<0.0001	<0.0001
Prediabetes to Diabetes					
MetS-IR		1.37 (1.32, 1.42)	1.35 (1.30, 1.41)	1.30 (1.24, 1.37)	1.26 (1.19, 1.33)
MetS-IR (quintile)					
Q1	259	Ref	Ref	Ref	Ref
Q2	401	1.62 (1.39, 1.90)	1.50 (1.28, 1.76)	1.48 (1.26, 1.74)	1.45 (1.23, 1.70)
Q3	495	1.95 (1.68, 2.27)	1.75 (1.50, 2.04)	1.62 (1.38, 1.90)	1.57 (1.34, 1.85)
Q4	556	2.22 (1.92, 2.57)	1.99 (1.71, 2.32)	1.79 (1.52, 2.11)	1.70 (1.44, 2.01)
Q5	713	2.82 (2.44, 3.25)	2.58 (2.23, 2.99)	2.19 (1.84, 2.60)	2.00 (1.68, 2.40)
P-trend		<0.0001	<0.0001	<0.0001	<0.0001

HR, hazard ratios; CI, confidence interval; other abbreviations as in **Table 1**.

Model I adjusted for age, sex, height, family history of diabetes, smoking status and drinking status.

Model II adjusted for age, sex, height, family history of diabetes, smoking status, drinking status, SBP, DBP, FPG, TG, HDL-C and LDL-C.

Model III adjusted for age, sex, height, family history of diabetes, smoking status, drinking status, SBP, DBP, FPG, TG, HDL-C, LDL-C. ALT, BUN and Cr.

### Dose-response relationship between MetS-IR and progression or regression of prediabetes

**Figure 4:**
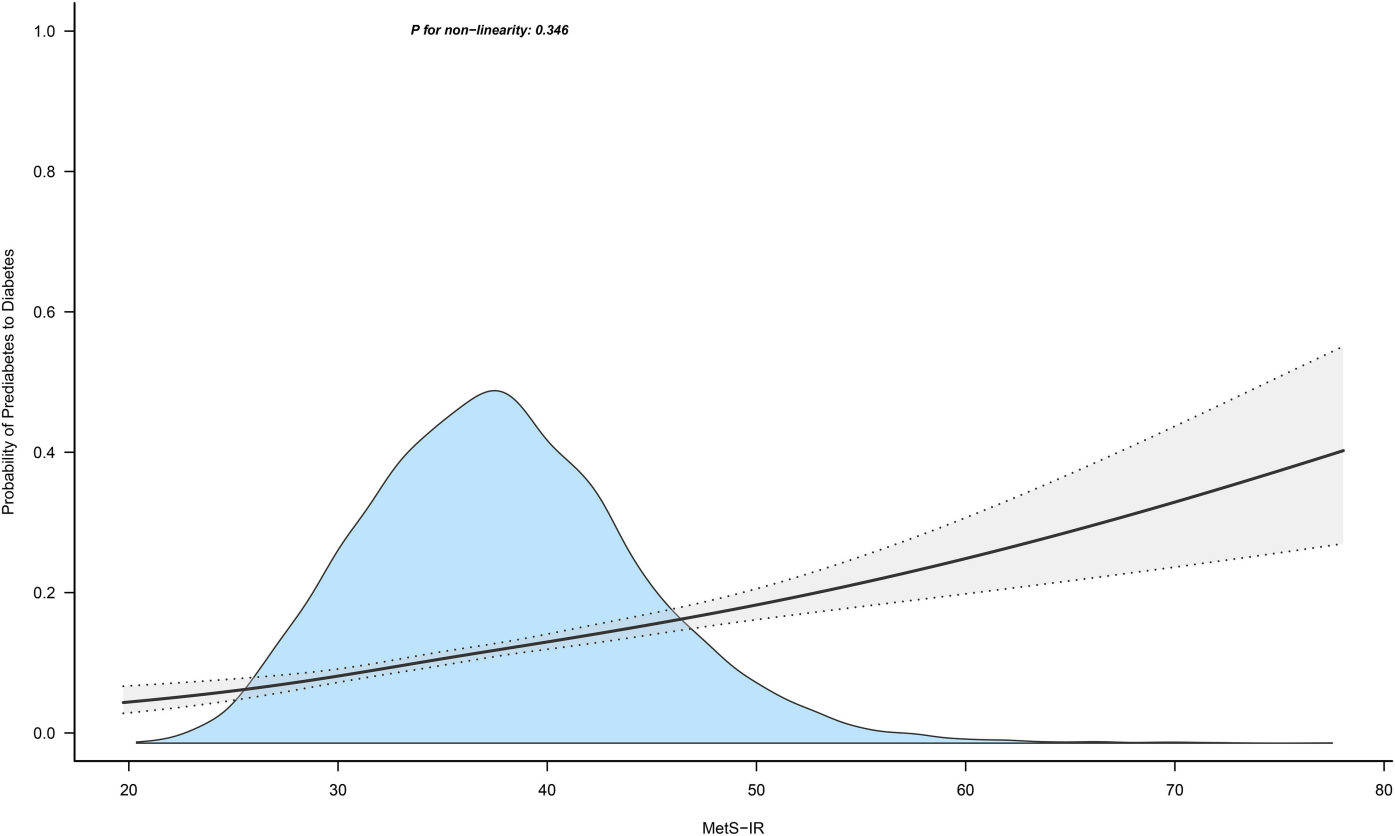
Apply the 4-knots RCS model to fit the dose-response curve of MetS-IR with the progression of prediabetes. Mets-IR, metabolic score for insulin resistance; RCS, restricted cubic splines.

**Figure 5:**
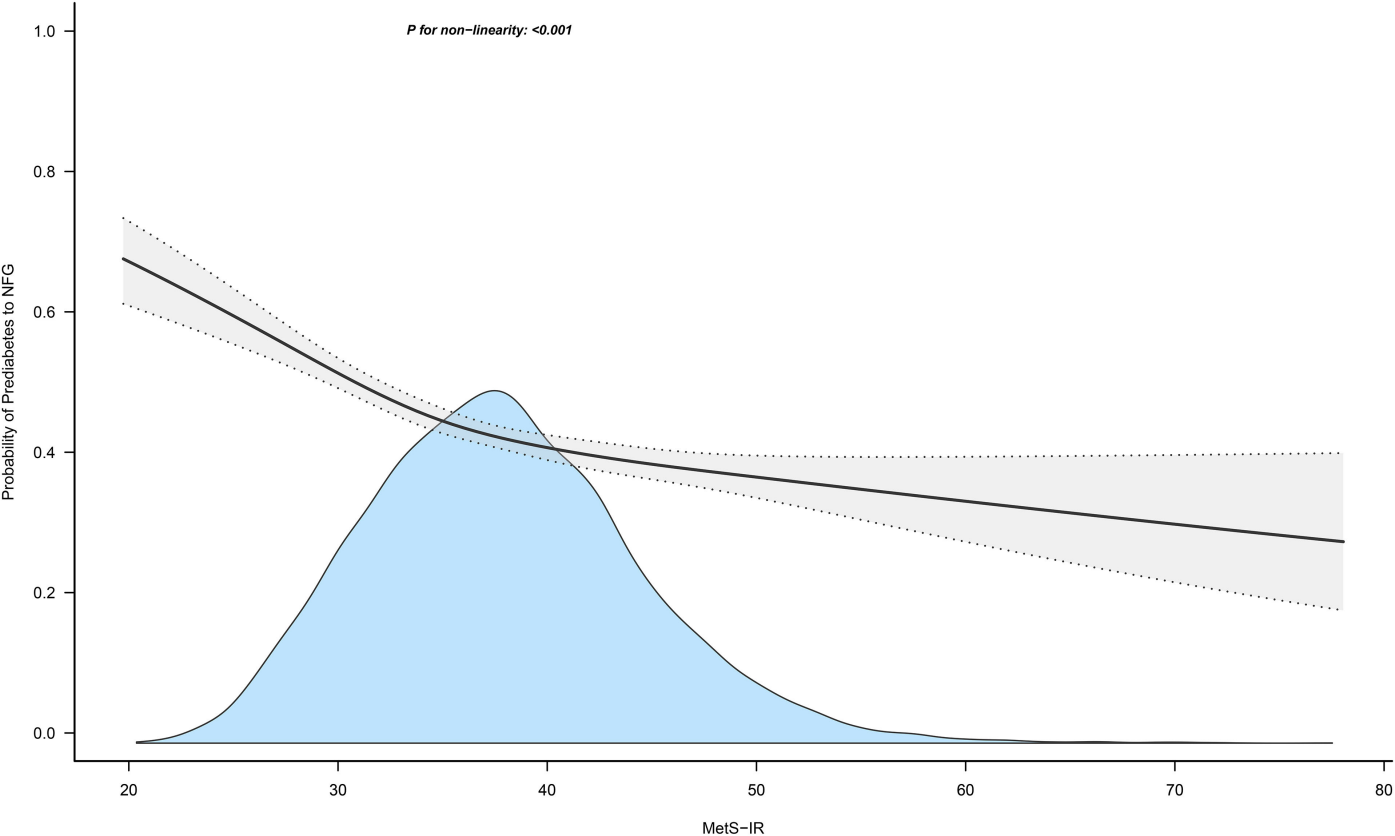
Apply the 4-knots RCS model to fit the dose-response curve of MetS-IR with the regression of prediabetes. Mets-IR, metabolic score for insulin resistance; RCS, restricted cubic splines; NFG, normal fasting glucose.

**Figures 4** and **5** show the dose-response curves of MetS-IR with the progression or regression of prediabetes. After multivariate adjustment based on Model III, we found a nonlinear correlation between MetS-IR and prediabetes regression (*P* for non-linearity < 0.001), and a linear correlation with prediabetes progression (*P* for non-linearity = 0.346). It was observed in the dose-response curve (**Figure 5**) that the recovery rate to NFG was higher when MetS-IR was lower, significantly dropping when MetS-IR reached a certain value (between 35-40) (*P* for log likelihood ratio test < 0.001). Using recursive algorithms and segmented regression, the inflection point for MetS-IR related to prediabetes regression was determined to be 37.22 (**Table 3**), with HRs of 0.95 (0.93-0.96) before and 0.98 (0.97-0.99) after the inflection point.

**Table 3:** The threshold effect of MetS-IR on the regression of pre-diabetes to NFG was analyzed by two-piecewise cox regression model.

Fitting model by two-piecewise cox regression	Adjusted HR (95%CI)	P-value
The inflection point of MetS-IR	37.22	
**< 37.22**	0.95 (0.93, 0.96)	<0.001
**> 37.22**	0.98 (0.97, 0.99)	0.003
P for log likelihood ratio test		<0.001

### Sensitivity analyses

Several sensitivity analyses were conducted, showing consistent results. First, treating MetS-IR as a categorical variable yielded consistent results (**Table 2**). Further analyses were repeated with changed inclusion criteria (WHO) and excluding participants with a family history of diabetes, showing no significant change in results (**Supplementary Table 3**). The Fine-Gray method considering competing risks was used, again yielding consistent results (**Supplementary Table 3**); finally, to verify the appropriateness of the 4-knot RCS in the current study, additional analyses using 3-knot and 5-knot RCS were performed, with no substantial changes in results (**Supplementary Figures 3**–**6**).

### Subgroup analysis

As shown in **Table 4**, within subgroups stratified by gender, age, and BMI, we observed a significant effect of the association between MetS-IR and prediabetes progression only in the age subgroup (*P* interaction < 0.05). In the age subgroup, compared to participants aged ≥45, those aged <45 had a stronger positive correlation between MetS-IR and prediabetes progression (HR: 1.42 vs 1.19, *P*-interaction < 0.0001).

**Table 4:** Exploratory subgroup analysis of the role and differences of MetS-IR in assessing changes in glycemic status in prediabetes patients.

	Prediabetes to NFG		Prediabetes to Diabetes	
	No. of case	HR (95%CI)	No. of case	HR (95%CI)
Gender				
Male	3949/10009 (39.45%)	0.90 (0.86, 0.94)	1702/10009 (17%)	1.25 (1.18, 1.33)
Female	2532/5412 (46.78%)	0.89 (0.85, 0.94)	722/5412 (13.34%)	1.27 (1.18, 1.38)
*P*-interaction		0.7820		0.6921
Age, years				
<45	2968/5327 (55.71%)	0.89 (0.84, 0.94)	544/5327 (10.21%)	1.42 (1.31, 1.53)
≥45	3513/10094 (34.80%)	0.89 (0.84, 0.94)	1880/10094 (18.62%)	1.19 (1.12, 1.26)
*P*-interaction		0.9344		<0.0001
BMI, kg/m^2^				
<24	3151/6261 (50.32%)	0.84 (0.77, 0.91)	709/6261 (11.32%)	1.52 (1.27, 1.83)
24-27.9	2504/6674 (37.52%)	0.84 (0.76, 0.93)	1138/6674 (17.05%)	1.35 (1.10, 1.65)
≥28	826/2486 (33.23%)	0.90 (0.81, 0.99)	577/2486 (23.21%)	1.23 (1.07, 1.43)
*P*-interaction		0.4679		0.1982

HR, hazard ratios; CI, confidence interval; other abbreviations as in **Table 1**.

Models adjusted for the same covariates as in model III (**Table 3**), except for the stratification variable.

## Discussion

In our study of 15,421 prediabetic subjects, we observed that an increase in the surrogate for IR, MetS-IR, was associated with an increased risk of developing diabetes, while a decrease in MetS-IR was protective in returning to NFG. Additionally, we found a nonlinear relationship between MetS-IR and the regression of prediabetes, with 37.22 identified as the inflection point; prediabetes regression rates were significantly higher before this point and markedly decreased thereafter.

Prediabetes is an early stage of diabetes and various chronic complications (1–4), in which IR is the main pathophysiological feature of the disease. To date, numerous studies have been undertaken globally to evaluate the effects of various intervention strategies on prediabetes, with non-pharmacologic (lifestyle adjustment) as well as pharmacologic methods (magnesium supplements, lipase inhibitor, glucagon-like peptide 1 receptor agonists, fenofibrate, alpha-glucosidase inhibitors, insulin sensitizer and traditional Chinese medicine) considered to have good potential for application (11, 17–21, 42). It is important to note that despite the large number of studies showing the effectiveness of pharmacologic approaches to treating prediabetes, no medications have been approved by regulatory agencies for the treatment of prediabetes, and expert statements only recommend lifestyle interventions as a first-line treatment for prediabetes (42, 43). In this context, it is important to monitor IR in the context of enhanced lifestyle interventions in prediabetic patients. Considering that HEGC, the gold standard for measuring IR, has major limitations, monitoring IR alternatives may have better application and promotion potential. As a new simple IR surrogate indicator, MetS-IR covers blood glucose, blood lipids, and obesity factors (27), and this parameter has been confirmed in recent studies to be the best IR surrogate for assessing diabetes risk in Chinese and Japanese populations (30). Therefore, evaluating the role of MetS-IR in glycemic outcomes among individuals with prediabetes in this study may offer valuable insights for future prevention and treatment strategies.

The association between MetS-IR and diabetes has been supported by evidence from various studies across different ethnicities. Overall, a positive correlation exists between MetS-IR and diabetes, with high MetS-IR identified as a significant risk factor for both the prevalence and incidence of diabetes (27–30, 44, 45), including in baseline non-diabetic subjects, non-obese individuals, and hypertensive patients. In our study, we further confirm the role of high MetS-IR as a risk factor for the development of diabetes; notably, unlike previous studies, our research specifically evaluates subjects with prediabetes. Specifically, among prediabetic participants, each unit increase in MetS-IR is associated with a 26% increase in the risk of developing diabetes within three years. Additionally, it is worth mentioning that recent evidence from a rural cohort study in China suggests that there may be no significant association between MetS-IR and diabetes (46). On reviewing this study, we noted that their sample size (n=1,205) and limited number of diabetes outcomes (n=97) might have affected their findings. When categorizing MetS-IR, each group’s sample size reduces further, which can weaken or nullify associations due to insufficient test power (47). Moreover, the inclusion of similar variables like BMI, waist circumference, and conventional lipids, which often have collinearity, might have confounded their results (48). Combining these findings, we lean towards MetS-IR being a significant predictor of diabetes across various ethnicities.

Another key finding was the negative correlation between MetS-IR and prediabetes regression, suggesting that lowering MetS-IR has a protective role in recovering to NFG of prediabetic individuals. This finding was consistent across multiple sensitivity analyses, confirming its stability and significance for prediabetes intervention. We further explored this relationship by plotting dose-response curves, revealing a nonlinear correlation between MetS-IR and prediabetes regression. The probability of returning to NFG was significantly higher when MetS-IR was lower, particularly below the inflection point of 37.22. To the best of our knowledge, these findings have not been previously reported and therefore could not be analyzed in the current study for comparisons between different races, populations. Considering our main results, maintaining MetS-IR below 37.22 is crucial for prediabetics to return to normal glucose regulation.

Previous reports suggested that prediabetes and diabetes predominantly occur in men, older individuals, and those who are overweight/obese (5, 6, 49, 50). Our stratified analysis by gender, age, and BMI showed similar trends, with higher proportions of men, older individuals, and those overweight/obese among those progressing to diabetes, and higher proportions of women, younger individuals, and non-obese individuals among those regressing from prediabetes (**Table 4**). However, a potentially contradictory finding was that younger individuals (<45 years) showed a stronger positive correlation between MetS-IR and prediabetes progression (HR: 1.42 vs 1.19, *P*-interaction < 0.0001) compared to those aged ≥ 45. This suggested that MetS-IR may be particularly effective in assessing diabetes risk in younger populations. The reasons for this unique finding are unknown, but some speculations might help explain it: (i) Overweight/obesity surveys in China from 1989-2011 show a rapid increase in obesity rates among young adults (18-39 years) (51). Given that obesity is a major cause of IR (52), this rapid weight gain could be a significant factor in the increased diabetes risk associated with MetS-IR in young people. (ii) The rapid development of China’s economy in recent decades has brought immense mental and psychological stress, particularly to the working population (mainly younger individuals), exacerbating IR and metabolic disorders (53, 54), thus increasing the diabetes risk associated with MetS-IR in this group. Additionally, the national family planning policy has led to a rapid decline in the workforce (55, 56), potentially impacting these trends.

### Study strengths and limitations

This study is noteworthy due to the following strengths: (i) Based on the multicenter health examination cohort of Rich Healthcare Group, this study benefits from a large sample size, enabling extensive sensitivity and subgroup analyses, enhancing the robustness of the results. (ii) This analysis is the first to report the association between MetS-IR and the regression of prediabetes, offering valuable insights for prediabetes intervention based on MetS-IR thresholds. Moreover, no previous studies have explored the relationship between MetS-IR and diabetes in a prediabetic population.

However, some inherent limitations must be acknowledged: (i) Participants were primarily from the Chinese population, necessitating further research to ascertain the extent to which these findings are generalizable to other ethnic groups. (ii) Due to the lack of oral glucose tolerance test data and HbA1c measurement data in the original dataset, the diagnosis of outcomes in this study relied solely on FPG, which may lead to some bias in the incidence rates of outcomes; considering the diagnostic shortcomings, current research evidence recommends promotion mainly in patients with impaired fasting glucose. It’s important to note that most prediabetes-related studies diagnose based on FPG (2), as large-scale epidemiological surveys typically involve large sample sizes, with only a small proportion undergoing oral glucose tolerance testing. (iii) This study is a secondary creation based on the multicenter health examination cohort of Rich Healthcare Group, and the covariates included in the regression models were based on available variables in the dataset, inevitably leading to residual confounding from variables not included in the dataset (57). (iv) Although the follow-up period of the current study is substantial (2-7 years), the average follow-up duration remains relatively short; longer follow-up studies are needed to further clarify the impact of MetS-IR on long-term blood glucose changes in prediabetic patients. (v) The current study lacks MetS-IR data during follow-up, necessitating future research to assess the impact of increases/decreases in MetS-IR on the progression/regression of prediabetes. (vi) The current study did not identify people who used antidiabetic drugs for indications other than diabetes, which may have partially affected the results.

## Conclusion

In conclusion, our study provides new evidence from a general population that an increase in MetS-IR may lead to an increased risk of diabetes in individuals with prediabetes; similarly, a decrease in MetS-IR enhances the protective effect for returning to NFG.

## Data Availability

The datasets presented in this study can be found in online repositories. The names of the repository/repositories and accession number(s) can be found in the article/**Supplementary Material**.
