# Supplementary material for: Association of metabolic score for insulin resistance with progression or regression of prediabetes: evidence from a multicenter Chinese medical examination cohort study
Source: Front Endocrinol (Lausanne). 2024 Nov 11;15:1388751. doi: 10.3389/fendo.2024.1388751 (PMC11589820; doi:10.3389/fendo.2024.1388751)
Supplement: Supplementary file 1 [file DataSheet1.docx]

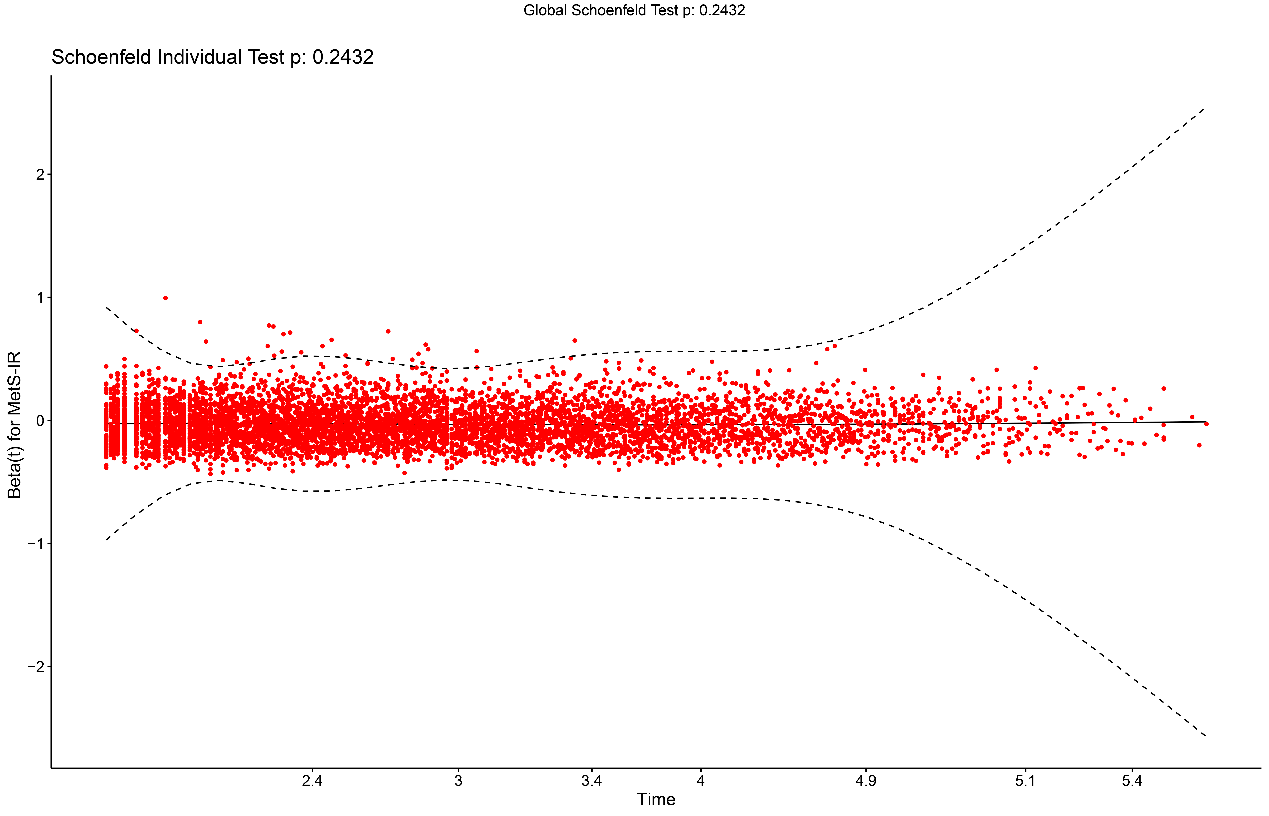


**Supplementary Figure 1**: Schoenfeld residual plot of MetS-IR changes over time with recovering from prediabetes to NFG as the dependent variable. The p-value of Schoenfeld Residuals Test result is larger than 0.05 which indicated that MetS-IR is not a time dependent variable and can be analyzed by Cox Proportional Hazards Model. Mets-IR: Metabolic score for insulin resistance; NFG: normal fasting glucose.


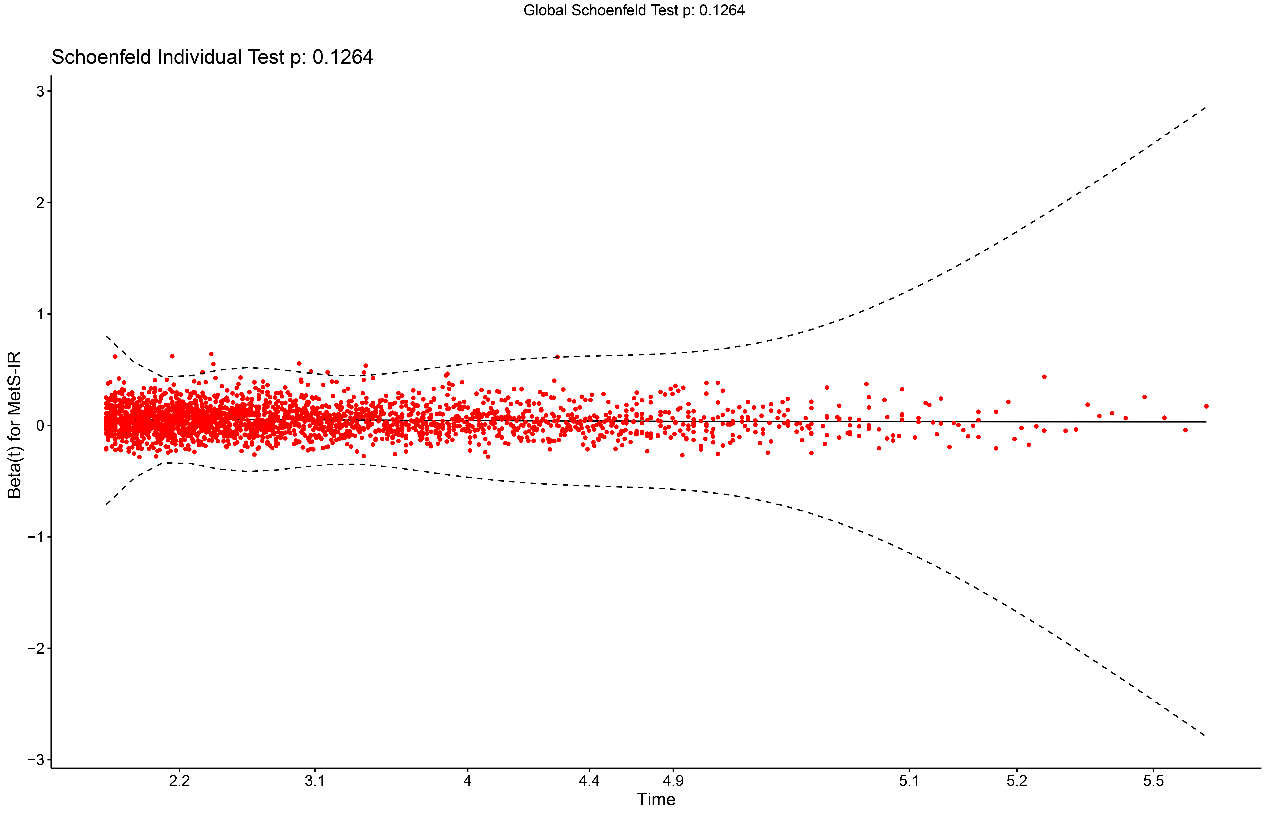


**Supplementary Figure 2:** Schoenfeld residual plot of MetS-IR changes over time with progression from prediabetes to diabetes as the dependent variable. The p-value of Schoenfeld Residuals Test result is larger than 0.05 which indicated that MetS-IR is not a time dependent variable and can be analyzed by Cox Proportional Hazards Model. Mets-IR: Metabolic score for insulin resistance.


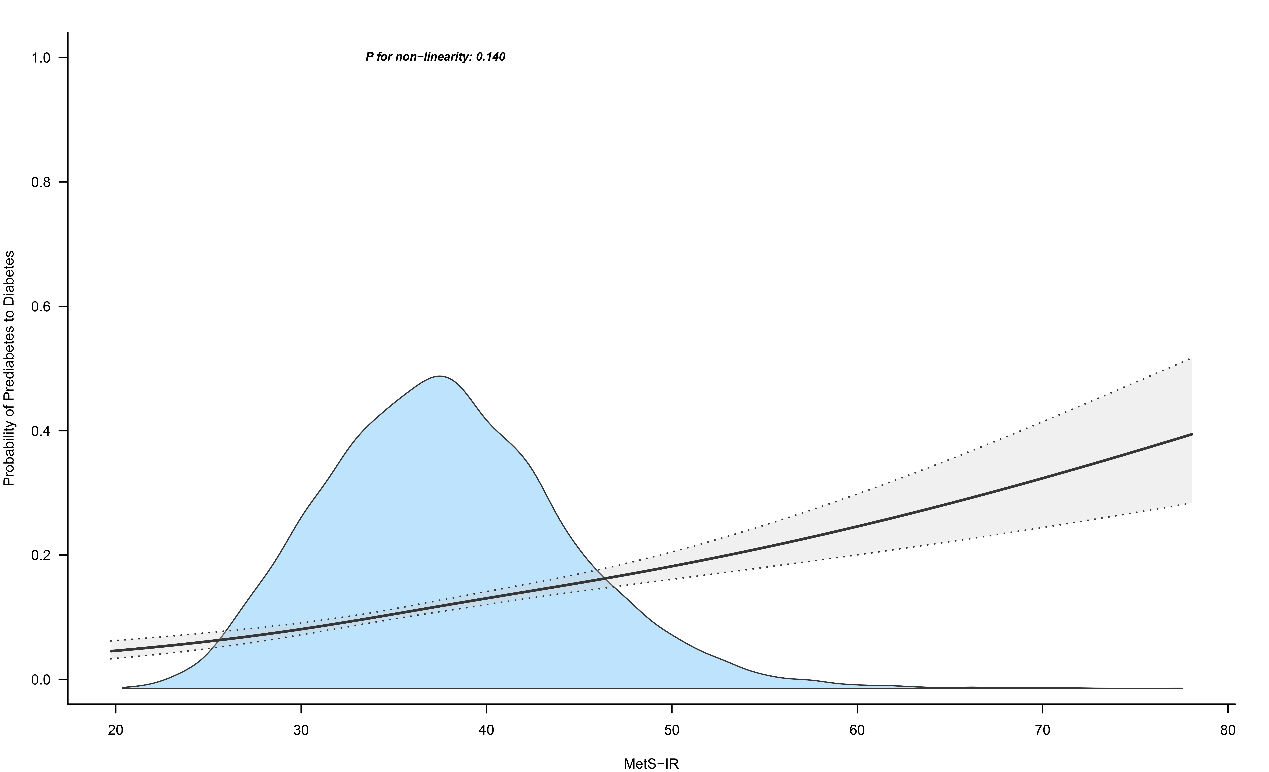


**Supplementary Figure 3**: Apply the 3-knots RCS model to fit the dose-response curve of MetS-IR with the progression of prediabetes. Mets-IR: Metabolic score for insulin resistance; RCS: restricted cubic splines.


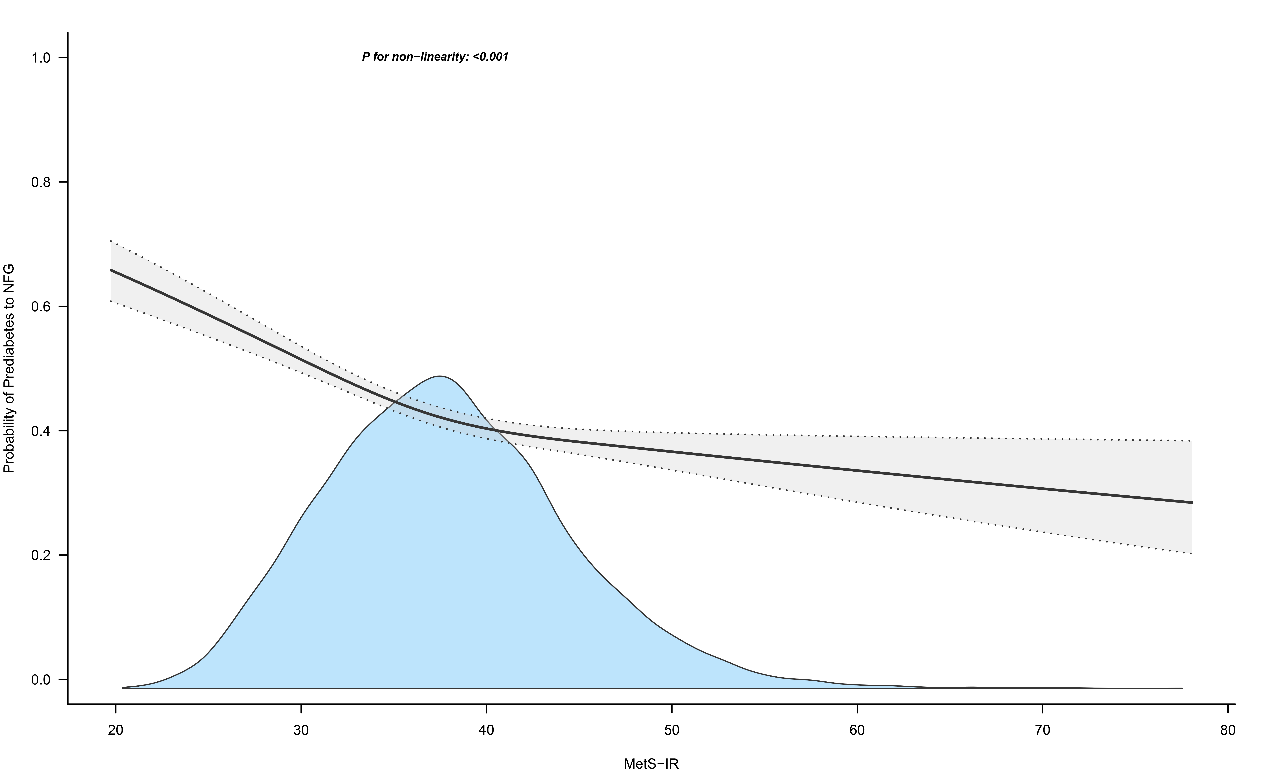


**Supplementary Figure 4**: Apply the 3-knots RCS model to fit the dose-response curve of MetS-IR with the regression of prediabetes. Mets-IR: Metabolic score for insulin resistance; RCS: restricted cubic splines; NFG: normal fasting glucose.


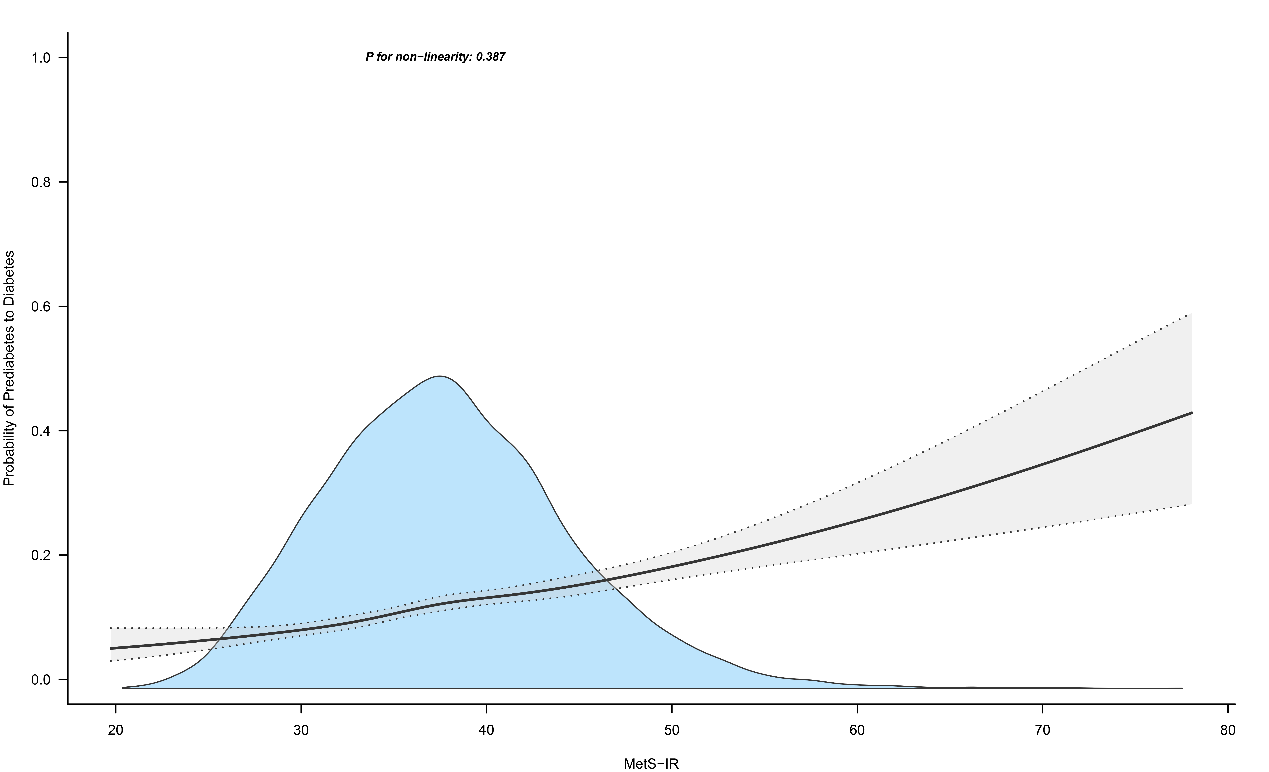


**Supplementary Figure 5**: Apply the 5-knots RCS model to fit the dose-response curve of MetS-IR with the progression of prediabetes. Mets-IR: Metabolic score for insulin resistance; RCS: restricted cubic splines.


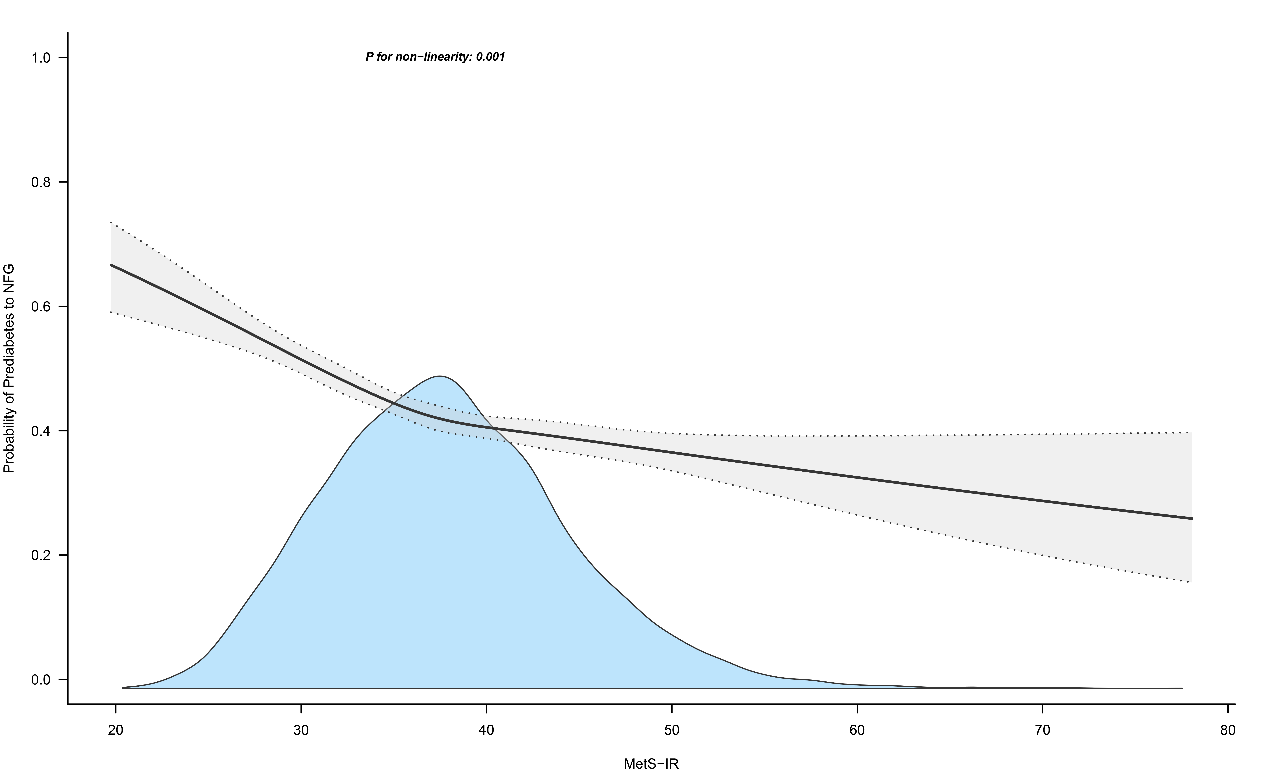


**Supplementary Figure 6**: Apply the 5-knots RCS model to fit the dose-response curve of MetS-IR with the regression of prediabetes. Mets-IR: Metabolic score for insulin resistance; RCS: restricted cubic splines; NFG: normal fasting glucose.
