# Supplementary material for: Association of metabolic score for insulin resistance with progression or regression of prediabetes: evidence from a multicenter Chinese medical examination cohort study
Source: Front Endocrinol (Lausanne). 2024 Nov 11;15:1388751. doi: 10.3389/fendo.2024.1388751 (PMC11589820; doi:10.3389/fendo.2024.1388751)
Supplement: Supplementary file 3 [file Table1.docx]

Supplementary Table 1: Diagnostic steps for collinearity between MetS-IR and other covariates when prediabetes is converted to NFG as the dependent variable.

|  | VIF | | | |
| --- | --- | --- | --- | --- |
|  | Step 1 | Step 2 | Step 3 | Step 4 |
| MetS-IR | 38.2 | 37.8 | 2.1 | 2.1 |
| Age | 1.4 | 1.4 | 1.4 | 1.4 |
| Sex | 2.6 | 2.6 | 2.6 | 2.6 |
| Height | 49 | 2.1 | 2.1 | 2.1 |
| Weight | 137.6 | NA | NA | NA |
| BMI | 93.6 | 22.9 | NA | NA |
| SBP | 1.9 | 1.9 | 1.9 | 1.9 |
| DBP | 1.7 | 1.7 | 1.7 | 1.7 |
| FPG | 1.1 | 1.1 | 1.1 | 1.1 |
| TC | 5.7 | 5.7 | 5.7 | NA |
| TG | 4 | 4 | 1.9 | 1.4 |
| HDL-C | 4.7 | 4.7 | 1.6 | 1.5 |
| LDL-C | 4.9 | 4.9 | 4.9 | 1.1 |
| ALT | 3.4 | 3.3 | 3.3 | 3.3 |
| AST | 3 | 3 | 3 | 3 |
| BUN | 1.1 | 1.1 | 1.1 | 1.1 |
| Cr | 1.7 | 1.7 | 1.7 | 1.7 |
| Family history of diabetes | 1 | 1 | 1 | 1 |
| Smoking status | 2.7 | 2.7 | 2.7 | 2.7 |
| Drinking status | 2.7 | 2.7 | 2.7 | 2.7 |

VIF: variance inflation factor; VIF = 1/(1-R^2^). Abbreviations as in Table ​1.

Note: The variables with VIF>5 will be regarded as collinear variables and cannot be included in the multiple regression model.

Supplementary Table 2: Diagnostic steps for collinearity between MetS-IR and other covariates when prediabetes is converted to diabetes as the dependent variable.

|  | VIF | | | |
| --- | --- | --- | --- | --- |
|  | Step 1 | Step 2 | Step 3 | Step 4 |
| MetS-IR | 38.2 | 37.8 | 2.1 | 2.1 |
| Age | 1.4 | 1.4 | 1.4 | 1.4 |
| Sex | 2.6 | 2.6 | 2.6 | 2.6 |
| Height | 49 | 2.1 | 2.1 | 2.1 |
| Weight | 137.6 | NA | NA | NA |
| BMI | 93.6 | 22.9 | NA | NA |
| SBP | 1.9 | 1.9 | 1.9 | 1.9 |
| DBP | 1.7 | 1.7 | 1.7 | 1.7 |
| FPG | 1.1 | 1.1 | 1.1 | 1.1 |
| TC | 5.7 | 5.7 | 5.7 | NA |
| TG | 4 | 4 | 1.9 | 1.4 |
| HDL-C | 4.7 | 4.7 | 1.6 | 1.5 |
| LDL-C | 4.9 | 4.9 | 4.9 | 1.1 |
| ALT | 3.4 | 3.3 | 3.3 | 3.3 |
| AST | 3 | 3 | 3 | 3 |
| BUN | 1.1 | 1.1 | 1.1 | 1.1 |
| Cr | 1.7 | 1.7 | 1.7 | 1.7 |
| Family history of diabetes | 1 | 1 | 1 | 1 |
| Smoking status | 2.7 | 2.7 | 2.7 | 2.7 |
| Drinking status | 2.7 | 2.7 | 2.7 | 2.7 |

VIF: variance inflation factor; VIF = 1/(1-R^2^). Abbreviations as in Table ​1.

Note: The variables with VIF>5 will be regarded as collinear variables and cannot be included in the multiple regression model.

Supplementary Table 3: Sensitivity analysis.

|  | No. of subjects |  | *P* value |
| --- | --- | --- | --- |
| Sensitivity-1 | 4,227 | HR per SD increase (95%CI) |  |
| Prediabetes to NFG |  |  |  |
| MetS-IR |  | 0.91 (0.83, 0.99) | 0.0468 |
| Prediabetes to Diabetes | |  |  |
| MetS-IR |  | 1.20 (1.12, 1.29) | <0.0001 |
| Sensitivity-2 | 15,421 | SHR per SD increase (95%CI) |  |
| Prediabetes to NFG |  |  |  |
| MetS-IR |  | 0.73 (0.69, 0.77) | <0.0001 |
| Prediabetes to Diabetes | |  |  |
| MetS-IR |  | 1.40 (1.31, 1.49) | <0.0001 |
| Sensitivity-3 | 14,611 | HR per SD increase (95%CI) |  |
| Prediabetes to NFG |  |  |  |
| MetS-IR |  | 0.89 (0.86, 0.93) | <0.0001 |
| Prediabetes to Diabetes | |  |  |
| MetS-IR |  | 1.26 (1.20, 1.33) | <0.0001 |

SHR, subdistribution hazard ratios; CI, confidence; other abbreviations as in Table 1.

Note 1: Models adjusted for the same covariates as in model III (Table 3).

Note 2: (1) Sensitivity-1: including 4,227 subjects according to WHO's diagnostic criteria for DM and IFG; (2) Results of Competing Risks Model Analysis (N=15,421); (3) sensitivity-3: excluding subjects with a family history of diabetes (N= 14,611).
